# Insulated *piggyBac* and FRT vectors for engineering transgenic homozygous and heterozygous eHAP cells

**DOI:** 10.1242/bio.061793

**Published:** 2025-10-01

**Authors:** Annabel Y. Minard, Stanley Winistorfer, Robert C. Piper

**Affiliations:** Department of Molecular Physiology and Biophysics, University of Iowa College of Medicine, Iowa City, IA 52242, USA

**Keywords:** Flp-In, Heterozygous cells, Haploid cells, Insulated cloning vectors, *piggyBac*, Transgenic cells

## Abstract

Transgene expression in eHAP cells, a haploid cell line commonly used to generate gene knockouts, is difficult due to its low transfection efficiency and poor expression of integrated transgenes. To enable simple and reliable transgene expression, we engineered insulated integrating plasmids that sustain high levels of transgene expression in eHAP cells, and that can be used in other cell lines. These vectors are compatible with FLP-FRT and *piggyBac* integration, they flank a gene-of-interest bilaterally with tandem cHS4 core insulators, and co-express nuclear-localized blue fluorescent protein for identification of high expressing cells. We further demonstrate that transgenic haploid eHAP cells can be fused to form transgenic heterozygous diploid cells. This method creates diploid cells carrying the transgenic material of the haploid progenitors and allows for engineering of cells with defined heterozygous genotypes. These tools expand the range of experiments that can be performed in eHAP cells and other cultured cells.

## Teaser

**Summary:** Techniques for stable transgene expression in eHAP cells by using cHS4 insulators and for creating heterozygous diploids by fusion of haploid cells.

## INTRODUCTION

Altering gene copy number is a useful strategy to investigate gene function and dosage sensitivity. Haploid cell lines, such as eHAP cells, are a particularly useful platform for this approach since disruption of a single allele is sufficient to generate a homozygous loss-of-function genotype (Llargués-Sistac et al., 2023; Essletzbichler et al., 2014). However, to support a wider range of functional studies, reliable transgene expression must also be possible in these cells; this is important for applications such as overexpression, complementation, and reporter assays. Additionally, while haploid cells are ideal for generating knockouts, they cannot be used to study heterozygous genotypes. Developing methods to reliably express transgenes and generate heterozygous cell lines would allow more flexible use of eHAP cells in functional genomic studies.

eHAP cells are a fully haploid fibroblast-like cell line. They are derived from near-haploid HAP1 cells (Essletzbichler et al., 2014), which are derived from an attempt to generate pluripotent stem cells from KBM7 cells (Carette et al., 2011). KBM7 cells are a near-haploid human chronic myelogenous leukemia cell line (Andersson et al., 1995). Because of their haploid nature, eHAP, HAP1 and KBM7 cells have been used extensively for genomic screens (Carette et al., 2011; Tchasovnikarova et al., 2015; Beusch et al., 2023). The haploid genome of eHAP cells, like HAP1 and KBM7 cells, spontaneously reverts to a diploid state, creating homozygous diploid cells, which are commonly used for phenotyping (Andersson et al., 1995; Beigl et al., 2020; Minard et al., 2023; Olbrich et al., 2017). In contrast, human genomes are heterozygous at many loci, with one copy of the genome inherited from the haploid gametes of each parent. Although many diseases are linked to heterozygous alleles, experimentally assessing their impact is difficult. Engineering heterozygous cells from diploid cells using standard CRISPR gene editing is challenging due to a loss of heterozygosity (Alanis-Lobato et al., 2021). New methods to overcome this limitation involve simultaneous delivery of HDR templates for both alleles (Carlson-Stevermer et al., 2020; Sconocchia et al., 2023). Alternatively, heterozygous cells could potentially be produced by fusion of congenic haploid eHAP cells that are engineered with different alleles. An advantage of this strategy is that alleles would not need to be rederived when comparing multiple allele combinations, which would also allow for a more consistent genomic background. In support of this strategy, heterozygous cells have been successfully generated by fusing haploid embryonic stem cells using hemagglutinating virus of Japan envelope particles (Sarel-Gallily et al., 2022). Another method of cell fusion is to use polyethylene glycol (PEG), this technique is commonly used to produce hybridomas for antibody production.

The feasibility and reliability of many experiments depend on uniform transgene expression. Transgenes can be introduced into cells by plasmid or viral vectors, or by CRISPR-Cas9 targeted gene-knock-in. CRISPR-Cas9 targeted gene-knock-in can suffer from low efficiency, although new strategies are emerging that are promising (Nam et al., 2025). Plasmid vectors offer several advantages over viral systems: they are cheaper to produce, more stable, safer to handle, and do not require the complex packaging systems or specialized facilities needed for viral production. Viral vectors also present additional limitations, including restricted tropism, potential toxicity, and, in the case of retroviruses, silencing from LTR elements that can undermine durable transgene expression. While transient plasmid transfection avoids these issues, it can be plagued by low transfection efficiency and results in highly variable expression levels due to differences in plasmid copy number from cell to cell. Stable integration is an alternative that can produce consistent and reliable expression (Kim and Eberwine, 2010; Stepanenko and Heng, 2017). Stable integration can be achieved using transposase systems such as *piggyBac* or Flp recombinase. Both systems involve transfection of cells with a recombinase and an expression plasmid engineered for integration. The expression plasmid contains a gene-of-interest (GOI), and DNA elements that stimulate the recombinase to integrate the GOI into the genome. With the Flp-In system, Flp-recombinase inserts a transgene into an Flp recombinant target (FRT) site previously introduced into the host's genome (O'Gorman et al., 1991; Craig, 1988; Sauer, 1994). Because integration occurs only at an FRT site, this method creates congenic stable cell lines. In contrast, *piggyBac*-mediated integration, does not require engineering an integration site but produces heterogenous integrations throughout the genome. The *piggyBac* transposase integrates transgenes flanked by inverted terminal repeats (ITR) into sites throughout the genome, with a bias for active chromosomal regions rich in AATT sequences (Ding et al., 2005; Liang et al., 2009; Galvan et al., 2009).

Integrated transgenes can suffer from silencing, meaning their expression is reduced (Cabrera et al., 2022). Silencing is often mediated by repressive covalent modifications on DNA and histones. These modifications promote the spreading of heterochromatin and repel transcription machinery (Greer and Shi, 2012; Greenberg and Bourc'his, 2019; Allshire and Madhani, 2018). The causes of silencing are not always known, but can include the sequence, genomic position, and integration method of the transgene, as well as the cell type of the transgene host (Cabrera et al., 2022). The most common and best understood strategy to overcome gene silencing is by using insulators. Insulators are *cis*-regulatory elements that possess both barrier and/or enhancer blocking activity, they exert their effects by recruiting the transcription factors CTCF, USF1 and VEZF1 (Yahata et al., 2007). A well-known barrier insulator is chicken hypersensitive site 4 (cHS4), from the chicken β-globin gene cluster (Raab and Kamakaka, 2010). The majority of the insulating effect of the cHS4 insulator is contained within a 250 bp ‘core insulator’ that recruits chromatin-modifying enzymes (Chung et al., 1997).

Here we describe methods to generate both transgenic and heterozygous eHAP cells. We found that the efficiency of transient transfection in eHAP cells was low, and stably integrated transgenes suffered from reduced transgene expression over time, potentially by gene silencing. We overcome this with two vectors for simple and reliable transgene expression in eHAP cells. These vectors improve transgene expression by 1) using the *piggyBac* or Flp-In System to stably integrate transgenes, 2) incorporating tandem cHS4 core insulators that diminish epigenetic silencing, and 3) co-expressing nuclear-localized blue fluorescent protein (BFP-NLS) to identify and sort for cells with transgene expression. The insulated expression vectors can also be used in other mammalian cell lines that suffer from gene silencing. We also demonstrate that transgenic haploid eHAP cells can be fused to create transgenic heterozygous diploid cells. This method creates diploids carrying the integrated vectors and genotypes of the haploid progenitor cells. In this method, isogenic haploid cells are fused via PEG and the resulting diploid cells can be selected for based on acquisition of antibiotic resistance and fluorescent protein markers from the haploid progenitors. This method provides an additional means of introducing transgenic vectors into eHAP cells, and also allows for the engineering of heterozygous genotypes.

## RESULTS

### An insulated FRT-expression vector for high levels of stable transgene expression

**Fig. 1.**
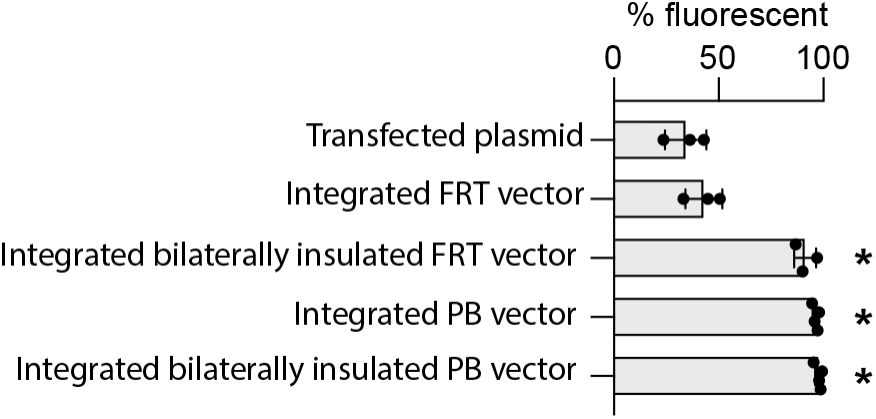
**Integration of insulated vectors increases the proportion of expressing cells.** The uninsulated FRT vector (without Flp recombinase) was transiently transfected into eHAP cells using lipofectamine, and fluorescence was measured 2 days later. The uninsulated and bilaterally insulated FRT vector were integrated into eHAP-FRT cells. The uninsulated and bilaterally insulated *piggyBac* vectors were integrated into eHAP cells. Fluorescence in eHAP cells with integrated vectors was assessed within 2 weeks after antibiotic selection. Fluorescence was measured by FACS (*n*≥3, *P<0.05). Data for integrated FRT vectors and *piggyBac* vectors are reproduced in Figs 2C and 3C, respectively.

**Fig. 2.**
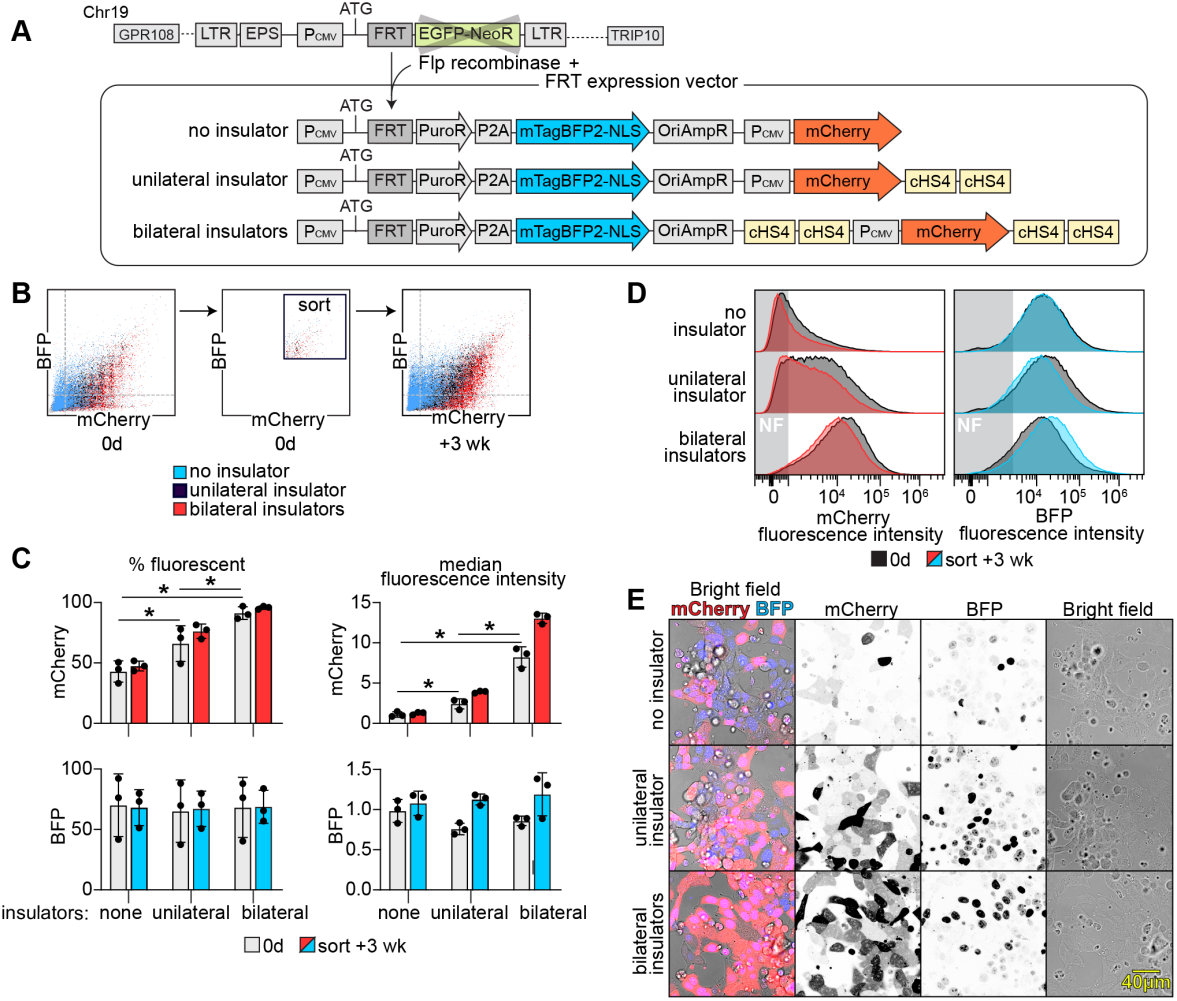
**Integration of the insulated FRT-expression vector enables high levels of stable transgene expression in eHAP-FRT cells.** (A) Schematic of the FRT locus in eHAP-FRT cells and the vectors used in this study. The FRT locus in eHAP-FRT cells is characterized by a CMV promoter, followed by an ATG site and FRT site. Once the vectors are integrated at the FRT site, Puro^R^-P2A-BFP-NLS is expressed from the genomic CMV promoter, whereas mCherry is expressed from a second CMV promoter contained within the vector. Tandem cHS4 core insulators are placed downstream of mCherry in the unilaterally insulated construct, whereas they are placed both upstream and downstream of mCherry in the bilaterally insulated construct. (B-E) The uninsulated and insulated FRT-expression vectors were integrated into eHAP-FRT cells. Within 2 weeks of antibiotic selection mCherry and BFP expression were assessed by FACS. Bright cells were isolated by FACS, passaged for 3 weeks, and fluorescence intensity was assayed again. (B) Shown is a scatter plot of the fluorescence intensities of mCherry and BFP from a representative experiment, after antibiotic selection, and after sorting for bright cells and passaging for 3 weeks. (C) Quantification of the % fluorescent cells and median fluorescence of cells analyzed in B (*n*=3, *P<0.05). (D) Histogram of the distribution of fluorescence intensities of the cells assayed in B. The region labeled ‘NF’ contains non-fluorescent cells. (E) Fluorescence microscopy of transgenic cells after antibiotic selection. Live cells were imaged on a confocal microscope.

We found that transient transfection of eHAP cells, by either reverse-transfection with lipofectamine or by electroporation (see the Materials and Methods for protocols), achieved a transfection efficiency of approximately 34% (Fig. 1) (Minard et al., 2023). To increase the level of transgene expression, we developed systems for stable integration, starting with the Flp-In system. This involved introducing an FRT site into eHAP cells for site-specific recombination of FRT-expression vectors carrying a GOI. An FRT site was introduced into random sites in the eHAP genome by transducing eHAP cells with the pQCXIP FRT-EGFP-Neo^R^ vector via lentivirus (Minard et al., 2023). As shown in Fig. 2A, this vector introduces a locus containing a cytomegalovirus (CMV) promoter driving the expression of EGFP fused to a neomycin resistance gene (EGFP-Neo^R^), with an FRT site located between the start codon and the EGFP-Neo^R^ ORF. Flp-mediated recombination of a pcDNA5 expression vector, shifts the EGFP-Neo^R^ out of frame, and in its place inserts an antibiotic resistance gene, in this case the hygromycin resistance gene (*Hyg^R^*), followed by a second CMV promoter that drives the expression of a GOI, in this case mCherry. We isolated multiple eHAP-FRT-EGFP-Neo^R^ clones and assessed their ability to Flp-in pcDNA5 FRT-expression vector. The pcDNA5 FRT-expression vector and Flp-recombinase were co-transfected into eHAP-FRT-EGFP-Neo^R^ clones, and cells that Flp'd-in pcDNA5 were selected for with hygromycin. Although multiple eHAP-FRT-EGFP-Neo^R^ cell lines produced hygromycin-resistant clones, only two expressed mCherry, and the level of expression was low. The brightest eHAP-FRT-EGFP-Neo^R^ cell line was selected for further experiments and eGFP was deleted from it using CRISPR-Cas9.

**Fig. 3.**
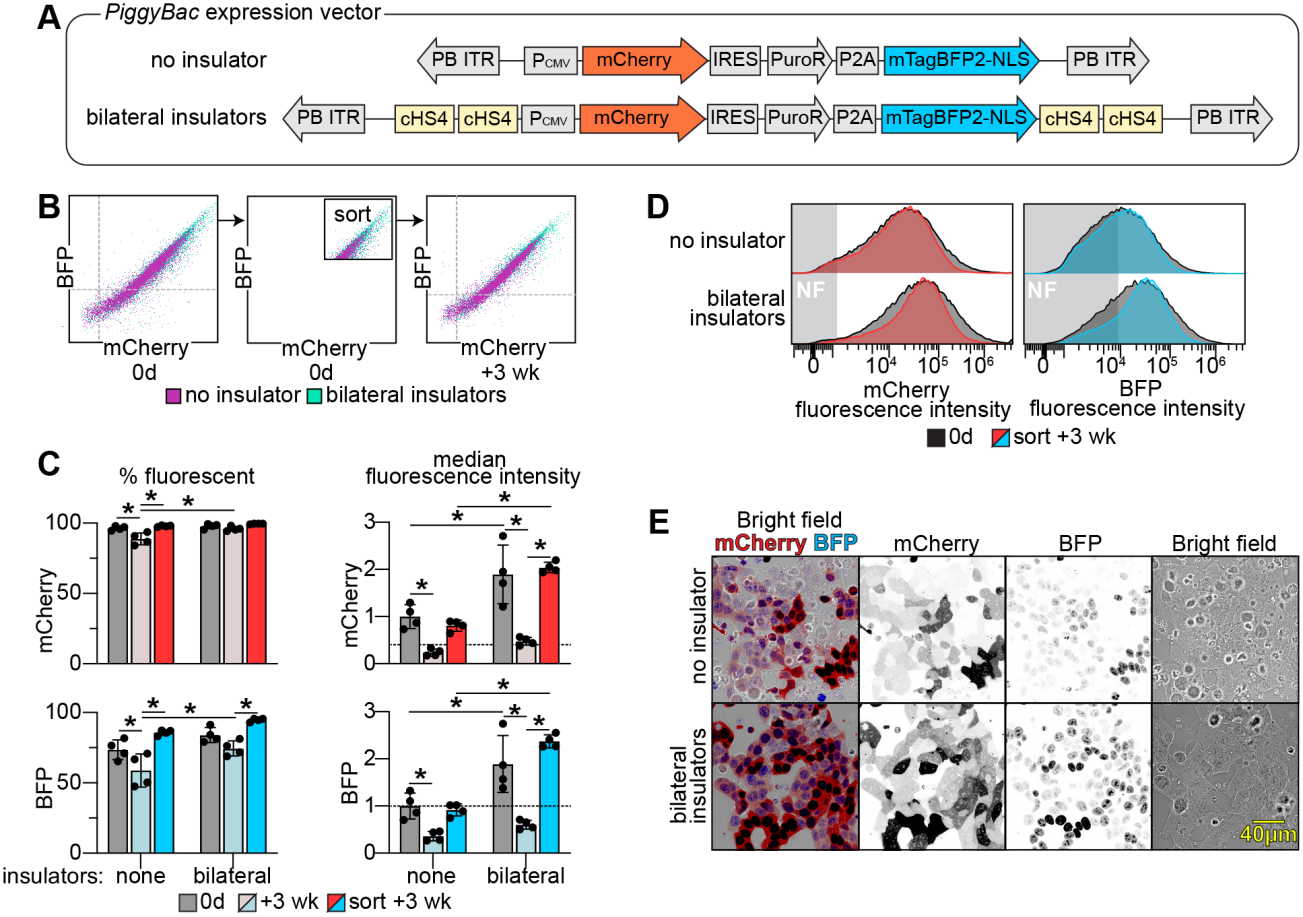
**Integration of the insulated *piggyBac*-expression vector enables stable expression of a transgene of interest in eHAP cells.** (A) Schematic of the uninsulated and insulated *piggyBac*-expression vectors used in this study. Both vectors express mCherry, Puro^R^, and BFP-NLS from a single CMV promoter, and are transcribed as separate proteins due to an IRES or P2A sequence separating the genes. The bilaterally insulated construct, but not the uninsulated construct, contains tandem cHS4 core insulators upstream and downstream of the ORF. (B-E) The uninsulated and insulated *piggyBac*-expression vectors were integrated into eHAP cells. Within two weeks of antibiotic selection mCherry and BFP expression were assessed by FACS. Bright cells were isolated by FACS, passaged for 3 weeks, and fluorescence intensity was reassessed. (B) Shown is a scatter plot of the fluorescence intensities of mCherry and BFP from a representative experiment, after antibiotic selection, and after sorting for bright cells and passaging for 3 weeks. (C) Quantification of the % fluorescent cells and median fluorescence of cells analyzed in B (*n*=4, *P<0.05). (D) Histogram of the distribution of fluorescence intensities from a representative experiment in B. NF=non-fluorescent cells. (E) Fluorescence microscopy of transgenic cells after FACS sorting for bright cells and passaging for 4 weeks. Live cells were imaged on a confocal microscope.

We examined if transgenes were recombining correctly at the FRT locus, by replacing Hyg^R^ in pcDNA5 with the puromycin-resistance gene fused to BFP with a nuclear localization signal (Puro^R^-P2A-BFP-NLS) (Fig. 2A). Expression of BFP-NLS indicates successful integration, since this ORF lacks a promoter and start codon in the plasmid and can express only from the genomic FRT site. Puro^R^ was used because it allows more rapid selection. Although we could observe BFP expression early after Flp-mediated integration, that fluorescence diminished over the course of a few days. This indicated that the FRT-expression vector had integrated, but gene expression was being reduced.

To overcome the observed reduction in transgene expression we modified the FRT-expression vector with tandem cHS4 core insulators (Yao et al., 2003; Chung et al., 1993). The cHS4 insulator possesses barrier activity that blocks the spreading of heterochromatin and repressive epigenetic modifications (Raab and Kamakaka, 2010). It also can block activity from nearby enhancers that might otherwise cause variable expression. In one vector we placed tandem cHS4 core insulators downstream of the mCherry cassette, and in another vector we placed tandem cHS4 core insulators both upstream and downstream of the mCherry cassette (Fig. 2A). The ORF encoding Puro^R^-P2A-BFP-NLS could not be bilaterally insulated using the vector, since it uses the CMV promoter encoded in the genome (Fig. 2A). We compared the level of mCherry expression achieved by integration of each vector using FACS (Fig. 2B). mCherry was expressed in 43% of cells without insulators, 66% with one set of tandem cHS4 core insulators, and 91% with bilateral tandem cHS4 core insulators (Fig. 2C,D). The median fluorescence of mCherry also increased 2.2-fold with unilateral insulators, and 7.5-fold with bilateral insulators. The expression of BFP-NLS was unaffected by the insulators; however, it remained a useful indicator of successful integration and a predictor of mCherry abundance. By microscopy, fluorescence of mCherry and BFP-NLS also improved from barely detectable levels of fluorescence in uninsulated constructs, to bright fluorescence from bilaterally insulated constructs (Fig. 2E).

Next, we compared gene expression over time from each of the constructs. Since the uninsulated construct exhibited low gene expression soon after integration, we sorted for cells that expressed a similarly high mCherry and BFP expression from each construct, passaged them for 3 weeks, and reassessed their gene expression (Fig. 2B). Expression from the uninsulated and unilaterally insulated constructs returned to low levels, whereas the bilaterally insulated construct expressed a higher level of mCherry with 96% of cells expressing mCherry and a 12-fold increase in median fluorescence relative that from the uninsulated construct after initial integration (Fig. 2C,D).

These results demonstrate that the bilaterally insulated FRT vector can be used to generate a high level of sustained expression of GOI. The inclusion of bilateral tandem cHS4 core insulators improved gene expression better than unilateral insulators. The bilaterally insulated construct increased fluorescence intensity 7.5-fold, and after enriching for high expressors, mCherry expression was sustained for 3 weeks and increased 12-fold. The benefits of the insulators were restricted to the mCherry locus, and did not benefit the Puro^R^-P2A-BFP-NLS locus.

### An insulated *piggyBac*-expression vector for stable expression of both a transgene of interest and BFP-NLS to monitor expression levels

We next sought to obtain high levels of stable gene expression using the *piggyBac* transposase system. We constructed a *piggyBac* expression vector that contained a CMV promoter driving the transcription of a GOI, in this case mCherry, followed by an IRES (internal ribosome entry site) that coupled the levels of mCherry translation with that of Puro^R^-P2A-BFP-NLS (Fig. 3A). The nuclear localized BFP is coupled to the expression of the GOI so that it can be used to monitor or select for the expression level of the population. This feature is useful because, unlike the Flp-In system, *piggyBac* transposase integrates transgenes into A/T-rich sites across the genome, each of which can influence expression differently. We generated versions of this construct with and without tandem cHS4 core insulators placed both upstream and downstream of the bicistronic cassette, as was done in the FRT vector (Fig. 2A). The vectors were co-transfected with a plasmid encoding *piggyBac* transposase into eHAP cells. A mixed population of puromycin resistant cells was selected for and their fluorescence intensity analyzed by flow cytometry.

The population of mCherry expressing cells in the uninsulated *piggyBac* vectors was higher than in the uninsulated FRT vectors, with 96% of cells expressing mCherry (Fig. 3B-E). This may be due to *piggyBac* vectors preferring to integrate at actively transcribed regions (Ding et al., 2005; Liang et al., 2009). The insulated *piggyBac* vectors improved gene expression further, with 98% of cells expressing mCherry, and a 1.7-fold increase in mCherry median fluorescence intensity relative to the uninsulated construct. BFP expression correlated tightly with mCherry expression in both constructs, which allows it to be used to track the abundance of the GOI (Fig. 3B,E). By microscopy we also observed that cells with the insulated construct had higher mCherry and BFP fluorescence compared to cells expressing the uninsulated construct (Fig. 3E).

To compare gene expression over time we enriched for similarly high expressing mCherry and BFP cells by FACS from both constructs, and reassessed gene expression after passaging the cells for 3 weeks (Fig. 3B). Compared to the start of the experiment, the median fluorescence of cells with the uninsulated construct decreased to 0.76-fold, whereas the insulated construct increased to 1.9-fold. We also assessed the median fluorescence of unsorted cells that were passaged for 3 weeks and found that the unsorted cells decreased 0.22-fold, and the insulated construct decreased to 0.44-fold in the unsorted group (Fig. 3C,D).

Thus, the insulated *piggyBac* vector improves expression of the GOI, in this case mCherry, 1.7-fold, and after enriching for high expressors, mCherry expression was sustained for over 3 weeks. In addition, BFP-NLS expression can be used to estimate mCherry expression levels.

### Engineering heterozygous diploids from fusion of transgenic haploids

During our experiments, and as noted in other studies, we found that eHAP cells spontaneously diploidized and generate mixed aneuploid populations (Beigl et al., 2020; Olbrich et al., 2017; Banerjee et al., 2022). We reasoned that directing diploidization in a controlled manner could provide an easy way to construct diploids of defined genotypes, such as heterozygous cells, or to introduce multiple GOIs into one cell line. In addition, we found that spontaneous diploids made from haploids are larger and have different morphological characteristics that lend themselves better to certain experiments (Minard et al., 2023). These morphological differences were examined in detail by another group (Beigl et al., 2020).

**Fig. 4.**
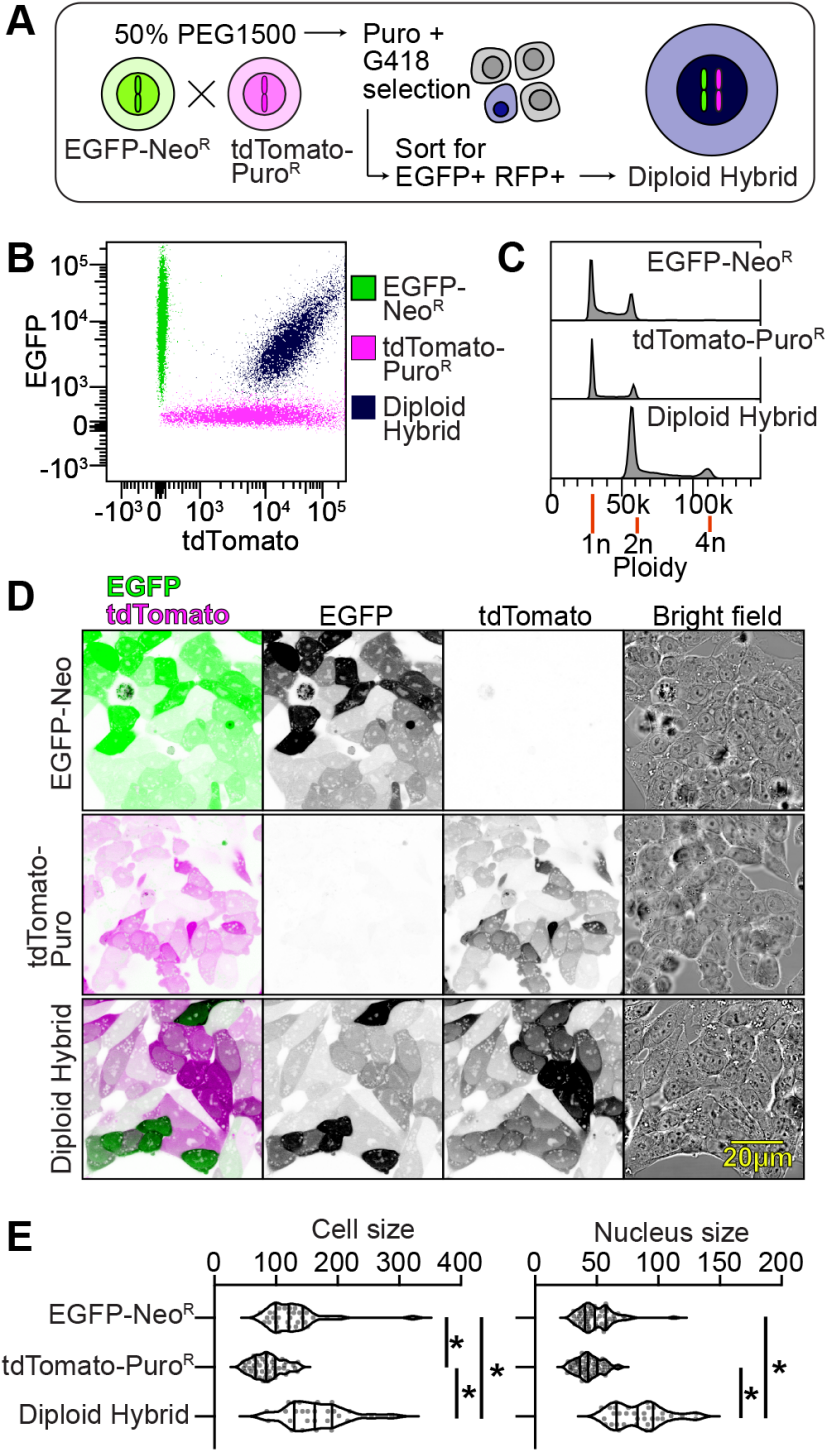
**Fusion of transgenic haploid eHAP cells produces heterozygous diploid eHAP cells.** (A) Schematic for haploid fusion. Haploid FRT-EGFP-Neo^R^ cells and haploid PB-tdTomato-Puro^R^ cells were fused with 50% PEG1500. Fused cells were selected for in puromycin and G418. Cells expressing both tdTomato and EGFP were isolated by disk cloning and enriched by flow cytometry. (B) EGFP and tdTomato fluorescence and (C) DNA content in haploid progenitors and diploid fusion cells. DNA content was assayed by PI staining and quantified using flow cytometry. (D) Fluorescence microscopy of haploid progenitors and the heterozygous diploid cell line was acquired on a confocal microscope. Overlay of EGFP and tdTomato is shown, as well as single channel images and bright field. (E) Violin plots of cell and nuclear size, based on cross-sectional area measured from fluorescence images in D (*n*=27-60 cells from a single experiment, *P<0.05).

To accomplish directed diploidization, we constructed transgenic haploid cells lines with different drug selection markers and different fluorescent markers. For one haploid cell line we used the haploid eHAP-FRT-EGFP-Neo^R^. For another, we used an eHAP cell line expressing Td-Tomato and Puro^R^ using the *piggyBac* system described above. We fused the cell lines using a method based on PEG, which has been used in other cell types (Yang and Shen, 2006). After incubation with PEG, we selected for fused hybrid cells using both G418 and puromycin (Fig. 4A). After isolating Neo^R^+/Puro^R^+ clonal cell lines, we performed flow cytometry analysis to verify which clones had acquired both EGFP and tdTomato from the haploid progenitors (Fig. 4B) and were diploid (Fig. 4C). Fig. 4 demonstrates the production of a hybrid diploid, with the predicted diploid DNA content, and co-expression of EGFP and tdTomato as observed by flow cytometry and microscopy. The cell and nucleus size are larger in the diploid population too.

**Fig. 5.**
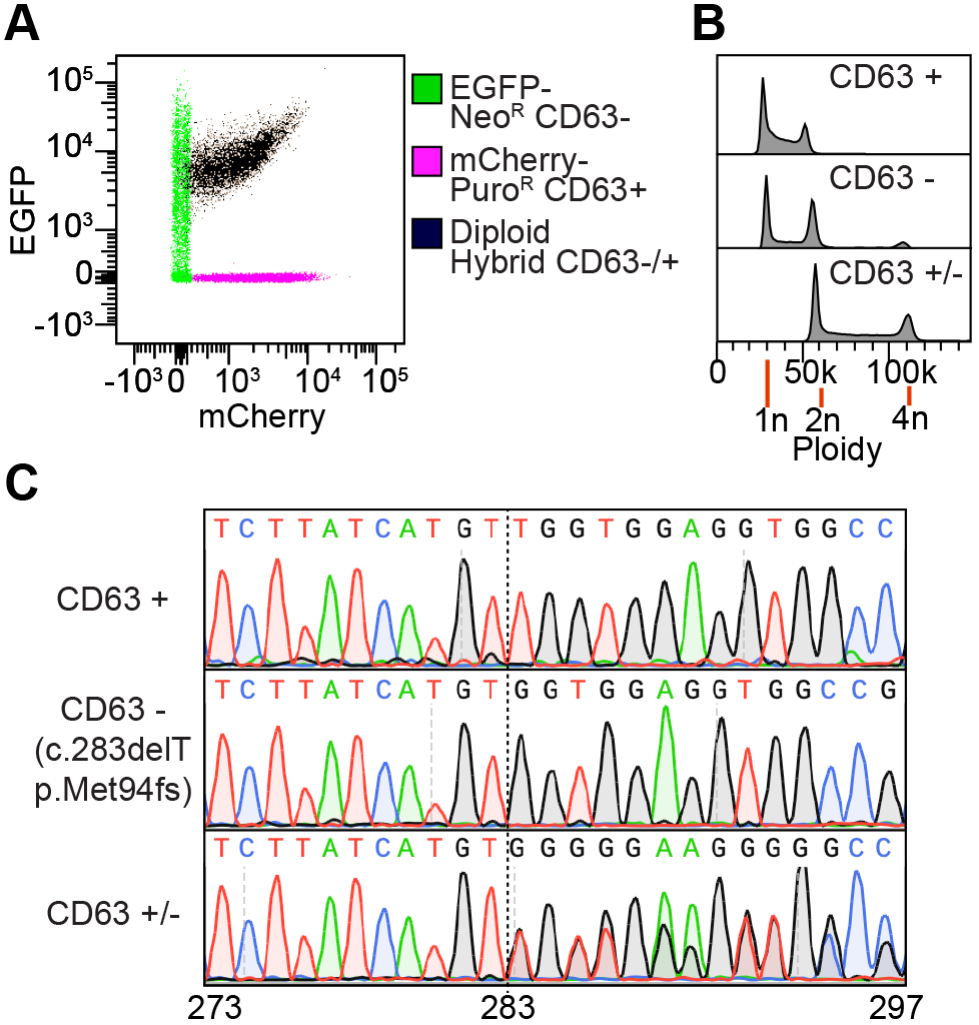
**Generation of CD63 heterozygous cells from fusion of transgenic haploid eHAP cells.** Haploid PB-EGFP-Neo^R^ CD63- cells and haploid FRT-mCherry-Puro^R^ CD63+ cells were fused with 50% PEG1500. Fused cells were selected for in puromycin and G418. Cells expressing both mCherry and EGFP were isolated by disc cloning and enriched by flow cytometry. EGFP and mCherry fluorescence (A) and DNA content (B) in haploid progenitors and diploid fusion is shown. EGFP and mCherry fluorescence was measured by flow cytometry. DNA content was assayed by PI staining and quantified using flow cytometry. (C) Sanger sequencing chromatogram of the CD63 locus in haploid progenitors and diploid fusion cells.

Next, we examined if we could engineer a cell with a heterozygous genotype using this method. The progenitor we used was a knockout CD63 cell line, which was generated by CRISPR-Cas9, that had the genotype c.283delT p.Met94fs. In the CD63- cells we integrated EGFP-Neo^R^ using an insulated *piggyBac* vector. This cell line was fused with a CD63+ haploid eHAP-FRT line with mCherry-Puro^R^ integrated from an insulated FRT vector. We isolated nine clones that expressed both EGFP and mCherry, four of which were diploid and two of which were triploid (data not shown). To test for heterozygosity at the CD63 locus, we performed Sanger sequencing on one diploid clone expressing both EGFP and mCherry (Fig. 5A,B), which is shown in Fig. 5. The chromatogram demonstrates overlapping base calls from c.283 onwards, consistent with a heterozygous c.283delT/wt (Fig. 5C).

Overall, these data demonstrate that haploid fusion can be used to construct heterozygous eHAP cells with the genotype and transgenic material of the progenitor cell lines.

## DISCUSSION

Here we describe insulated vectors and methods for the creation of transgenic homozygous and heterozygous eHAP cells. The insulated vectors enabled high levels of stable transgene expression in eHAP cells and can also be used in other cells. The vectors stably integrate transgenes via the site-selective Flp-recombinase or *piggyBac* transposase, contain flanking tandem cHS4 core insulators to sustain transgene expression, and co-express nuclear-localized BFP to monitor transgene expression. The vectors were assembled from existing components. Utilizing these vectors, we engineered diploid heterozygous cell lines by fusing transgenic haploid cells carrying complimentary fluorescent and antibiotic selection markers. This technique provides a solution to the challenge of engineering heterozygous cells, which is complicated by loss of heterozygosity with standard CRISPR-Cas9 approaches (Alanis-Lobato et al., 2021) and is useful for modelling compound heterozygous and polygenic disease-associated variants.

Both insulated *piggyBac* and Flp-In systems supported high levels of stable transgene expression, but each offers distinct features that may make one more suitable than the other depending on the experimental context. The Flp-In system requires a pre-engineered FRT site but enables the generation of congenic cells with the same transgene insertion site. In contrast, *piggyBac* does not require prior genome engineering, but integrates randomly. For this reason, we typically avoided clonal isolation when introducing a *piggyBac* construct into two different cell lines; however, if further genetic modifications are planned, isolating a clone beforehand is advisable to ensure a consistent genetic background. Because Flp-In integrates at a single defined FRT site, clonal isolation is typically unnecessary.

The insulated vectors were necessary because eHAP cells exhibited a pervasive and rapid loss of gene expression. Reduced transgene expression is a common problem in cell lines. The particular mechanism for loss of transgene expression in eHAP cells was not investigated here. Gene silencing commonly involves histone deacetylation followed by histone and DNA methylation (Cabrera et al., 2022; Mutskov and Felsenfeld, 2004) and can be triggered by the sequence (e.g. presence of CpG islands), genomic position (e.g. which influences susceptibility to encroachment of heterochromatin), integration method of the transgene (which could activate viral and transposon defense systems), proliferation rate (which could cause antagonism between replication and transcription machinery), and cell type (they have varying levels of gene silencing machinery) (Cabrera et al., 2022). In our experiments the FRT integration vector experienced more severe silencing than the *piggyBac* integration vector. This may be because the FRT integration site was introduced by lentivirus and lentiviral sequences are well-documented to activate silencing (Ramezani et al., 2003; Ramezani and Hawley, 2010). It could also be that *piggyBac* integrates in more actively transcribed regions of the genome (Ding et al., 2005; Liang et al., 2009).

At present there are only a few approaches for overcoming gene silencing, among which are insulators (Yahata et al., 2007). We employed tandem core cHS4 insulators and found that bilaterally placed insulators around the transgene substantially increased the level and durability of expression as compared to unilaterally placed insulators. Although the cHS4 insulator was effective in eHAP cells, they are not universally useful for overcoming all types of gene silencing. In some cell lines, cHS4 insulators in *piggyBac* vectors were reported to be beneficial (Mossine et al., 2013; Sharma et al., 2012), while in others no benefit was observed (Lu et al., 2020). Alternative insulators include the scaffold or matrix attachment region (S/MAR) or the ubiquitous chromatin opening element (UCOE). Novel insulators with comparable activity to the cHS4 insulator are also being discovered from CTCF binding sequences or mammalian-wide interspersed repeats (Cabrera et al., 2022; Liu et al., 2015; Groth et al., 2013; Zhang et al., 2023). To better predict when insulators are effective, some groups are performing massively parallel reporter assays. Surprisingly, a comparison of the cHS4, A2, ALOXE3 insulators across 10,000 locations in the genome of K562 cell line, revealed that only ALOXE3 acted as a heterochromatin barrier. cHS4 and A2 were found to block enhancers, and where they increased gene expression, were proposed to act as enhancers themselves (Hong et al., 2024). However, since this previous study used one full length cHS4 insulator on one side of the GOI, different effects might be observed with the dual flanking core cHS4 insulators used here.

Aside from insulators, other approaches to relieve epigenetic repression have been developed. One is to use histone deacetylase inhibitors (Zhang et al., 2019), although since these drugs exert global effects, it may not be appropriate for every experiment. Another is to disrupt the activity of the HUSH complex. The HUSH complex was identified by CRISPR mutagenesis to mediate silencing of a lentiviral reporter in KBM7 cells, a near haploid myeloid leukemia cell line, from which HAP1, and eHAP cells are derived (Tchasovnikarova et al., 2015). HUSH recognizes the absence of introns, and thus reintroduction of introns can prevent silencing by this complex (Seczynska et al., 2022). Another approach is to use synthetic chromatin regulators, to edit the epigenome of engineered loci (Park et al., 2019). As efforts to overcome gene silencing continue, alternative methods may prove to be more effective that the cHS4 insulators used here. In particular, it would be desirable to further reduce the variation in gene expression.

We also demonstrated that haploid eHAP cells can be fused to produce heterozygous diploid cell lines with defined genotypes. However, with this method it is important to isolate clones and check their ploidy, since it can produce triploids, probably from fusion of a haploid and diploid, and potentially tetraploids, from fusion of two diploids. The recovery of such clones could be minimized by starting with as pure a haploid population as possible. There are several applications of haploid fusion. This includes to introduce multiple expression vectors into a host, which is useful since the *piggyBac* and Flp-In system only support integration of one expression vector each per cell line. For example, although in our experiments we fused a *piggyBac* and FRT transgenic line, two FRT or two *piggyBac* transgenic haploid lines could be fused provided each had contrasting antibiotic and fluorescent markers. Another application is for haploinsufficiency screens, as demonstrated in a recent study using haploid embryonic stem cells (Sarel-Gallily et al., 2022), these screens can help assess when copy number variation is pathogenic. Thirdly, haploid fusion can be used to model compound heterozygote and polygenic disease-associated variants. This is useful given that many diseases are associated with heterozygous and polygenic genotypes but lack cellular models to assess their phenotype. Haploid fusion as means of engineering heterozygous cells is advantageous, as it does not suffer from the loss of heterozygosity, which occurs during CRISPR-cas9 gene editing of diploid cells, and allows for multiple allele combinations to be compared without re-deriving each variant (Alanis-Lobato et al., 2021).

In conclusion we have presented methods for effectively generating transgenic eHAP cells and for making heterozygous diploid eHAP cells. The methods here expand the experiments that are possible to conduct in eHAP cells.

## MATERIALS AND METHODS

### Cell culture

Human haploid eHAP cells (cat. # C669, Horizon Discovery, Cambridge, MA, USA) were cultured in Iscove's Modified Dulbecco's Medium (IMDM; Gibco, Billings, MT, USA) supplemented with 10% FCS. Cells were passaged every 48-72 h. Because eHAP cells can diploidize (Beigl et al., 2020), ploidy was checked and haploid cells enriched as necessary, which was generally every 3-4 weeks. For antibiotic selection of stable transfectants, eHAP cells were grown in IMDM FCS with puromycin 500 µg/ml and/or neomycin 500 µg/ml.

### Cell lines

eHAP-FRT-EGFP-Neo^R^ cells were generated as described previously (Minard et al., 2023). Briefly, eHAP cells were transduced with lentivirus carrying the pQCXIP FRT-EGFP-Neo^R^ (pPL6490) vector, which introduced the locus illustrated in Fig. 2A. eHAP-FRT cells were generated by CRISPR-Cas9 deletion of EGFP from eHAP-FRT-EGFP-Neo^R^ cells. Request for these cells lines can be made to Robert Piper. eHAP-CD63KO cells were generated by CRISPR-Cas9 deletion of CD63 from eHAP-FRT cells.

### CRISPR-Cas9 gene deletion

For CRISPR-Cas9 deletion, plasmids encoding Cas9 and gRNA were transiently transfected into eHAP-FRT cells. The plasmid pU6-(BbsI)_CBh-Cas9-T2A-mCherry encoded Streptococcus pyogenes Cas9 and mCherry (Chu et al., 2015). gRNA targeting EGFP (AAGTTCAGCGTGTCCGGCGA, GAGCTGGACGGCGACGTAAA, AGCACTGCACGCCGTAGGTC) were encoded in pU6-(BbsI) CBh-Cas9-T2A-mCherry. gRNA targeting CD63 (TCTGTCTCTTATCATGTTGG) was introduced into pCLIP dual SFFV ZsGreen (Transomics). 24 h after transfection cells were reseeded, 48 h after transfection mCherry fluorescent cells were isolated by fluorescence-activated cell sorting (FACS), serially diluted into 100 mm plates and allowed to form colonies. Colonies were isolated using trypsin-soaked discs (Domann and Martinez, 1995), which were placed into fresh dishes where cells were allowed to proliferate. Their ploidy was determined by Propidium Iodide staining. EGFP deletion was assessed by EGFP fluorescence. CD63 deletion was assessed by PCR amplifying the CD63 genomic locus using primers (CTTCCGGATGGGGTCAAGTC / CAAGAACCCAGCAACTTCGC), and Sanger sequencing the PCR product with a primer (GTCTTCCTCTTCCTGGTGGC).

### Plasmids for Flp-recombination and *piggyBac* integration

Flp-Recombinase Expression Vector pOG44 was from ThermoFisher. A hyperactive *piggyBac* transposase (Yusa et al., 2011) in a pcDNA3.1 vector was used as described in Ko et al. (2022). The uninsulated FRT integration vector, unilateral cHS4 FRT integration vector, bilateral cHS4 FRT integration vector, uninsulated *piggyBac* vector, and bilateral cHS4 *piggyBac* vectors were constructed for this study. These vectors were constructed using Gibson assembly (New England Biolabs, Ipswich, MA, USA), with intermediate constructs generated as needed. DNA inserts were PCR amplified for cloning using the NEBNext high fidelity enzyme master mix (New England Biolabs, Ipswich, MA, USA). DNA templates were sourced as follows: mCherry was PCR amplified from Addgene (#64324) (Chu et al., 2015), P2A-BFP-NLS was PCR amplified from a geneblock (IDT, Coralville, IA, USA), puromycin resistance cassette was PCR amplified from pQXCIP, tandem core cHS4 elements were amplified from Addgene plasmid (#58541) (Rival-Gervier et al., 2013). The backbone for the FRT vector was pcDNA5 (ThermoFisher, Waltham, MA, USA) and the backbone for the *piggyBac* vector was synthesized by Bio Basic (Ontario, Canada). The vector maps are provided in Supplemental Dataset 1. The vectors were sequenced using long-reading sequencing at plasmidsaurus (Eugene, OR, USA). The bilaterally insulated FRT (#217970) and *piggyBac* vector (#217971) were deposited in Addgene (Watertown, MA, USA).

### Plasmid transfection

Transfection of eHAP cells was performed by ‘reverse-transfection’ using Lipofectamine (Erfle et al., 2004). Compared to standard-transfection, reverse-transfection achieved a higher efficiency while requiring half the amount of reagent and one day less of preparation. In our best protocol, the DNA-lipofectamine mixture was prepared by mixing 1 µg DNA, 2 µl Lipofectamine 3000, 2 µl P3000 reagent, and 112 µl Opti-MEM, according to the manufacturer's instructions (Invitrogen/ThermoFisher, Waltham, MA, USA). The DNA lipofectamine mixture was combined with 6×10^6^ trypsinized cells, 2 ml of IMDM and 10% FCS in a single well of a six-well dish and incubated overnight. The following day cells were reseeded. Transient reverse transfection of the uninsulated FRT vector, without Flp recombinase, resulted in 34% of eHAP cells expressing RFP (Fig. 1).

Electroporations of eHAP cells were performed using the Lonza 4D Nucleofector. This system comes with three electroporation solutions and fifteen electroporation programs. Using the manufacturer's Cell Line Optimization Kit, we determined that the best protocol combined the SF solution with EH-100 program. If using the other solutions, the SE solution paired best with DS-120 or E0-100 programs, and the SG solution paired best with EH-100 program.

### Generation of stable cell lines by Flp-recombination or *piggyBac* integration

For Flp-recombination, pOG44 and FRT vectors were mixed at a 1:1 ratio and co-transfected into eHAP-FRT cells or eHAP-FRT-EGFP-Neo^R^ cells. For *piggyBac* integration, hyperactive *piggyBac* transposase and *piggyBac* vectors were mixed at a 1:1 ratio and co-transfected into eHAP cells. 24 h post-transfection cells were reseeded, 48 h post-transfection, both transfected cells and untransfected control cells were placed into the appropriate antibiotic selection medium. Within 2 weeks after controls cells died, the stable cell lines were assessed for fluorescence. To evaluate the stability of gene expression, high-expressing cells were enriched and passaged for 3 weeks before reanalysis.

### Karyotyping eHAP cells

**Fig. 6.**
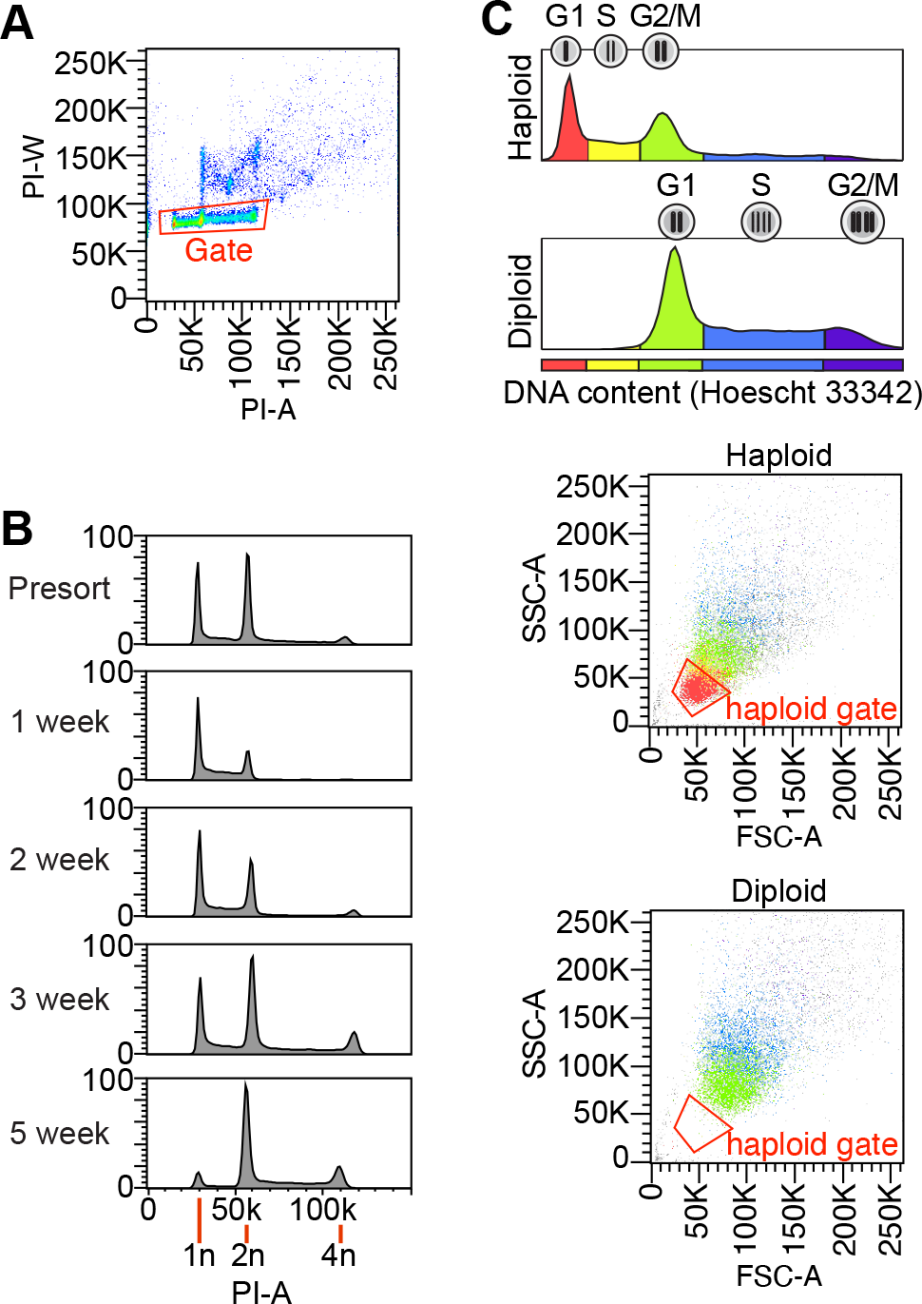
**Monitoring and maintaining eHAP ploidy by FACS.** (A,B) DNA content of eHAP cells stained with Propidium Iodide, analyzed by FACS. (A) Gates for single cells are drawn from plots of PI-A/PI-W. (B) Distribution of DNA content before enrichment of haploid cells by flow sorting, and several weeks after. DNA copy-number is indicated. (C) Gating strategy for enrichment of haploid cells by FACS. Haploid and diploid cells were stained with Hoechst 33342 to measure DNA content, and Hoechst 33342 fluorescence (top), FSC and SSC (bottom) of cells were measured by flow cytometry. The color of each data point corresponds to DNA content. Gate for sorting haploid cells is drawn around the G1 haploid population.

The ploidy of eHAP cells was determined by Propidium Iodide staining. Cells were trypsinized, washed twice with PBS, permeabilized and stained using Nicoletti buffer (0.1% sodium citrate, 0.1% Triton X-100, 0.5 unit/ml RNase A, 20 units/ml RNase T1, 50 μg/ml propidium iodide). The intensity of Propidium Iodide staining was measured on a Becton Dickinson LSR II flow cytometer. Gates for singlets were drawn as in Fig. 6A from PI-A/PI-W, and height of the peak corresponding to G1 haloid (1n) was monitored to assess the haploid population Fig. 6B. Haploid eHAP cells were used for reference.

### Enriching haploid eHAP cells

Haploid cells were enriched by FACS for cells with low forward scatter area (FSC-A) and side scatter area (SSC-A) (Fig. 6C). The gate used for sorting haploid cells was based on FSC-A and SSC-A and drawn to exclude diploid cells as shown in Fig. 6C. The approach was validated by monitoring the FSC-A and SSC-A profiles of cells stained with the nuclear DNA dye Hoechst 33342 (cat. #H3570, ThermoFisher, MA, USA) (Fig. 6C). Furthermore, Propidium Iodide staining of sorted cells confirmed haploids were enriched by the procedure (Fig. 6B). Haploid cells were enriched every 3-4 weeks of passaging, since after 5 weeks of passaging haploid cells are depleted and need to be enriched (Fig. 6B).

### Haploid fusion

Heterozygous diploid cells were produced by fusing two haploid eHAP cell lines expressing contrasting fluorescent and antibiotic selection markers. In one experiment we fused haploid eHAP-FRT-EGFP-Neo^R^ cells and haploid PB-tdTomato-Puro^R^ cells. In a second experiment we fused haploid eHAP FRT-mCherry-Puro^R^ cells and eHAP-CD63KO-PB-EGFP-Neo^R^ cells. The protocol for cell fusion was adapted from the protocol by Yang and Shen (2006). Haploid eHAP cells were grown to 50% confluency in a 10 cm dish. The cells were harvested by trypsinization, resuspended in IMDM FCS, pelleted at 100 ***g*** for 5 min and resuspended in 5 ml IMDM without serum. 1 ml of resuspended cells from both cell lines were mixed, pelleted at 100 ***g*** for 5 min, resuspended in 50% PEG 1500 (cat. #10783641001, Roche, Indianapolis, IN, USA) and incubated for 2 min at room temperature. To this reaction, 10 ml IMDM was added, and incubated for 20 min at room temperature. Cells were pelleted at 100 ***g*** for 5 min and resuspended in IMDM FCS and plated onto a 3.5 cm dish. The following day, cells were trypsinized and serially diluted in IMDM FCS with 500 ng/ml puromycin and 1 mg/ml neomycin and plated onto 10 cm dishes. After cells multiplied into colonies, a colony expressing both EGFP and tdTomato was isolated using disc cloning and expanded. EGFP and tdTomato expressing cells were enriched for by flow cytometry, expanded, karyotyped and genotyped.

### FACS

For cell sorting experiments, a Becton Dickinson Aria Fusion or a Cytek Aurora CS equipped with a 130 µM diameter nozzle was used. FACS data was analyzed using Flow Jo Version 10 (BD Biosciences) software. The gating strategy to measure fluorescence is shown in Fig. S1. Cells were identified based on FSC-A/SSC-A, singlets were identified based on FSC-A/FSC-W (Fig. S1). Fluorescent cells were identified by their separation from a non-fluorescent reference population, which was included in every experiment.

### Microscopy

eHAP cells were seeded at ∼10-20% confluency on glass-bottom dishes (MatTek, P35G-1.5-20-C) and imaged 36-48 h later, without changing culture media, using a Leica SP8 confocal microscope equipped with 405, 488 and 552 nm lasers, with emission bands of 30 nm centered at 457 nm, 520 nm, and 610 nm for BFP, EGFP, and mCherry, respectively. Images within the same experiment were acquired under the same settings. To quantify cell and nuclear surface area, three fields of view were recorded in a single experiment, ROI were manually defined and the area quantified using Fiji.

### Statistical analysis

Statistics were performed using GraphPad Prism 10 on data collected from at least three independent biological replicates, except Fig. 4E, which is one biological replicate. Investigators were not blinded to samples. Data were compared using one-way ANOVA with Šidák correction for multiple comparisons. Data were expected to have normal distributions and equal variances.

## Supplementary Material

10.1242/biolopen.061793_sup1 Supplementary information
