# Supplementary material for: Insulated piggyBac and FRT vectors for engineering transgenic homozygous and heterozygous eHAP cells
Source: Biol Open. 2025 Oct 1;14(10):bio061793. doi: 10.1242/bio.061793 (PMC12519548; doi:10.1242/bio.061793)
Supplement: Supplementary information [file biolopen-14-061793-s1.pdf]

## Dataset 1. Plasmid maps of *piggyBac* and FRT vectors

GenBank files (.gbk) containing annotated sequences of the *piggyBac* and FRT vectors used in this study. The files can be opened with SnapGene Viewer.

**Dataset DOI:** 10.6084/m9.figshare.29936843

**File: mCherry_FRT_PuroR_P2A_mTagBFP2_NLS.gbk**
**Description:** Uninsulated FRT vector

**File: mCherry--2xcHS4_FRT_PuroR_P2A_mTagBFP2_NLS.gbk**
**Description:** Unilaterally insulated FRT vector

**File: 2xcHS4--mCherry--2xcHS4_FRT-PuroR-P2A-mTagBFP2-NLS.gbk**
**Description:** Bilaterally insulated FRT vector

**File: PiggyBac_mCherry-IRES-PuroR-P2A-mTagBFP2-NLS.gbk**
**Description:** Uninsulated *piggyBac* vector

**File: PiggyBac_2xcHS4--mCherry-IRES-PuroR-P2A-mTagBFP2-NLS--2xcHS4.gbk**
**Description:** Bilaterally insulated *piggyBac* vector

Available for download at
https://journals.biologists.com/bio/article-lookup/doi/10.1242/bio.061793#supplementary-data

Biology Open • Supplementary information

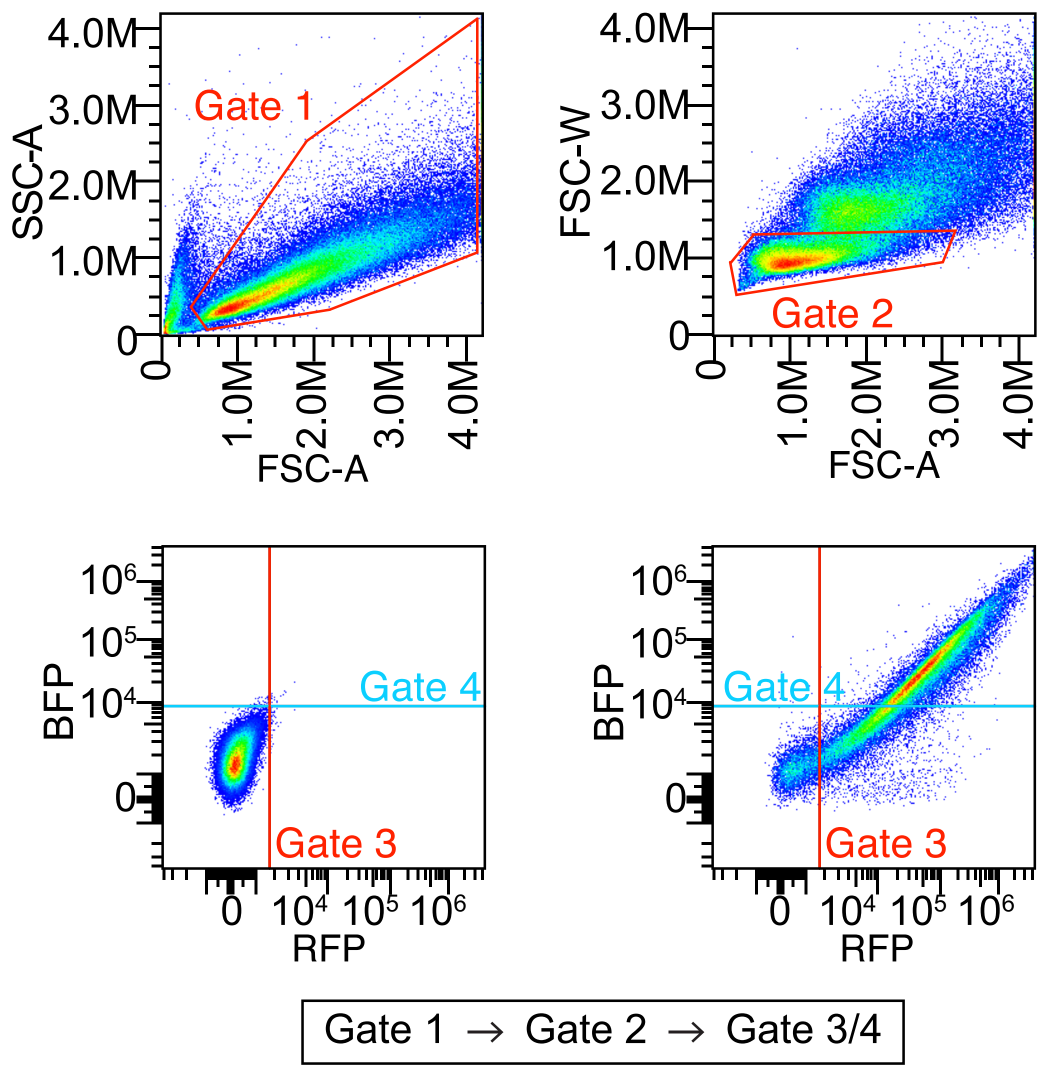


**Fig. S1. FACS gating for measuring fluorescence**

**A)** Gating strategy for measuring fluorescence of eHAP cells. Gates forsingle cells are drawn from FSC-A/SSC-A, followed by FSC-A/FSC-W. Fluorescent cell gates are drawn around non-fluorescent cells

Biology Open • Supplementary information
